# Fluorescence Quenching as a Diagnostic Tool for Prediction Reliability Assessment and Anomaly Detection in EEM-Based Water Quality Monitoring

**DOI:** 10.1021/acs.est.5c05952

**Published:** 2025-09-05

**Authors:** Yongmin Hu, Céline Jacquin, Eberhard Morgenroth

**Affiliations:** † 28499 Eawag: Swiss Federal Institute of Aquatic Science and Technology, 8600 Dübendorf, Switzerland; ‡ ETH Zürich, Institute of Environmental Engineering, Zürich 8093, Switzerland; § Gujer AG, 8153 Rümlang, Switzerland

**Keywords:** water quality monitoring, EEM, fluorescence quenching, outlier detection

## Abstract

Excitation–emission matrix (EEM) spectroscopy offers rapid and informative water monitoring, but its reliability is limited by chemical composition variability, which disrupts the relationship between fluorescence signals and contaminant concentrations. Recognizing this limitation, the lack of a robust and physically interpretable tool for assessing prediction reliability has become a critical bottleneck. In this work, the composition and photophysical inconsistencies among fluorescent compounds underlying the same fluorophore signal were identified as key sources of predictive inaccuracy. To detect these inconsistencies, fluorescence quenching was incorporated into EEM analysis with parallel factor analysis (PARAFAC). Apparent *F*_0_/*F*—the ratio of PARAFAC component intensity before and after extrinsic quencher addition—was proposed as an indicator for model failure and treatment anomaly detection. Validations with both model compound mixtures and real-world greywater samples showed that shifts in apparent *F*_0_/*F* reflect changes in the relationship between *F*_max_ and target concentrations of total cell count (TCC) and dissolved organic carbon (DOC). Two practical tools were developed based on apparent *F*_0_/*F*: a clustering method for post hoc chemical composition analysis, and a thresholding method for outlier detection in real-time monitoring. This work highlights the added value of fluorescence quenching for improving the reliability and interpretability of EEM-based water monitoring at the subfluorophore level.

## Graphical abstract

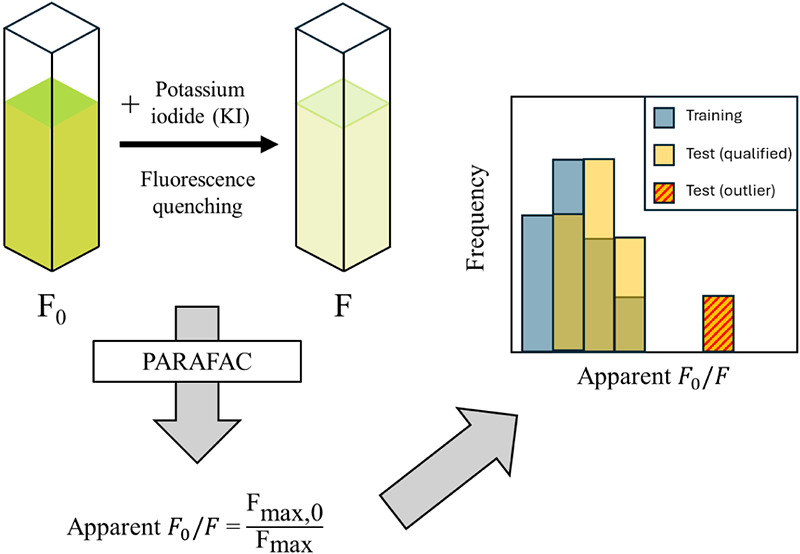

## 1 Introduction

Fluorescence spectroscopy with excitation–emission matrix (EEM) has gained increasing attention in environmental monitoring for its ability to rapidly detect a wide range of fluorescent organic compounds in water samples.^1–4^ Naturally, EEM signals are contributed by various fluorophores, i.e., the chemical moieties that show fluorescence.^5–9^ In this light, decomposing EEM using parallel factor analysis (PARAFAC) has become the standard approach for extracting representative signals using PARAFAC components with the advantage of better interpretability over black-box data-driven models.^10–12^ Globally, components with highly similar spectra recur across diverse water sources, reflecting the capture of fluorescent compounds with potentially similar bases of fluorophores.^6,13^

However, PARAFAC has two major limitations. First, distinguishing compounds with highly overlapping but yet slightly shifted spectra is inherently difficult due to the statistical constraints that limit the number of interpretable components.^14–17^ Second, the assumption that emission spectra are independent of excitation wavelength may not reflect the spectral shift in some dissolved organic matter (DOM) caused by intramolecular or intermolecular energy transfer.^18–20^ Therefore, a PARAFAC component should be interpreted as a generalized and simplified representation of compounds sharing chemically similar fluorophores with *F*_max_ (maximum intensity of a PARAFAC component) reflecting the combined contribution of these compounds to fluorescence intensity. Since PARAFAC components do not exactly represent either total organic matter or specific target compounds, it is an inherently biased proxy for estimating water quality parameters. Accurate quantification requires a stable quantitative relationship between *F*_max_ and the target. These relationships reflect mechanistic associations between the proxy compounds and the target (e.g., coremoval or competitive removal during treatment) but can break down under chemical composition variability, including the emergence of compounds with highly similar spectra but different mechanistic relevance to the target,^21,22^ or increased intrinsic quenching, which lowers fluorescence per unit of proxy concentration.^23^ This limitation has been demonstrated in previous studies investigating the quantification of general dissolved organic matter,^24–26^ bacteria,^21^ micropollutants,^10,27,28^ and disinfection byproducts.^29^ In these cases, parameters of the predictive models using *F*_max_ exhibited substantial variabilities from different water sources or the same source but at different temporal scales. Particularly, our earlier work found that even with minimized spectral shift in a component, the quantitative relationship between its *F*_max_ and contaminant concentrations (e.g., bacteria and different molecular weight fractions of DOM) varied significantly.^21^ The lack of generalizability highlights the need for system-specific calibrations when using EEM-PARAFAC; however, even with tailored models, robust performance is not guaranteed. The absence of a reliable and physically interpretable prediction reliability assessment tool remains one of the most critical barriers undermining faith in EEM-based water monitoring in practice.

Since the compositional heterogeneity among spectrally similar compounds cannot be resolved solely through *F*_max_, incorporating an additional parameter capable of distinguishing among these compounds at the subcomponent level is necessary. Previous studies on model compounds have shown that perturbations in pH,^18,30^ temperature,^31,32^ and fluorescence quenching agents (e.g., iodide, heavy metals, humic substances)^23,33,34^ can provide insights into distinguishing fluorescent compound compositions. From a practical perspective, dosing a fluorescence quencher is a more empirical approach as it is easy to implement in online monitoring and is sensitive to composition changes. For compounds with a similar fluorophore basis, their sensitivity to quenching can differ due to molecular structural features such as the extent of fluorophore exposure, conformational flexibility, and microenvironments around fluorophores.^35–38^ This sensitivity is captured by the quenching ratio (*F*_0_/*F*), defined as the ratio of fluorescence intensity in the absence of a quencher (*F*_0_) to that in the presence of a quencher (*F*). In ideal conditions, their relationship follows the Stern–Volmer equation:^39^

1 $$\frac{F_{0}}{F}=1+K\left[Q\right]$$

where [*Q*] is the quencher concentration, and *K* is the Stern–Volmer quenching constant. While deviations from this simple linear form often occur depending on the specific quenching mechanism involved, the general principle holds that *F*_0_/*F* is a function of compound-specific properties if the quencher type and concentration are fixed. With EEM, it is not possible to measure the *F*_0_/*F* of every independent fluorescent compound due to limited data decomposition resolution, but it is possible to characterize the apparent *F*_0_/*F* for a PARAFAC component using the component intensity indicator *F*_max_:

2 $$\mathrm{apparent}\;F_{0}/F=F_{\max,\mathrm{original}}/F_{\max,\mathrm{quenched}}$$

where *F*_max,original_ is the *F*_max_ in the original sample, and *F*_max,quenched_ is the *F*_max_ after dosing a specific concentration of an extrinsic quencher to the original sample. The apparent *F*_0_/*F* serves as a generalization of the *F*_0_/*F* of all fluorescent compounds underlying a component and may reveal compositional and photophysical changes involving these compounds. The purpose of this work is to propose and validate apparent *F*_0_/*F* as a novel indicator for abnormal composition diagnosis and unreliable prediction detection in EEM-based water monitoring. Specifically, the following questions were addressed:

What factors cause changes in apparent *F*_0_/*F*, and how do these changes correspond to shifts in the quantitative relationship between *F*_max_ and target contaminant concentrations? Does apparent *F*_0_/*F* remain effective in the presence of intrinsic quenchers, such as humic substances?
Is the value of apparent *F*_0_/*F* physically interpretable? Does it reflect the share of spectrally overlapping compounds with distinct *F*_0_/*F*?
How can apparent *F*_0_/*F* be applied in model failure and system anomaly detection for water treatment monitoring, especially in real-time scenarios? What value does it add beyond other fluorescence indices and numerical error metrics?

## 2 Material and Methods

### 2.1 EEM Measurement and Preprocessing

EEMs were measured with an Aqualog fluorescence spectrometer (HORIBA, Japan). A thermostat was used to control the cuvette temperature at 20 °C. The excitation wavelength ranged from 274 to 400 nm with a 2 nm interval, and the emission wavelength ranged from 309.6 to 500.4 nm with a 1.19 nm interval. Each sample was measured twice: the first EEM was measured with the original sample, and the second EEM was measured after adding potassium iodide (KI) to the sample. KI was selected as the quencher for three reasons. First, it is nonfluorescent, so it did not introduce additional fluorescence signals in EEM. Second, the absorbance of KI is negligible above 274 nm, so the additional inner filter effect was negligible. Third, it is a low-toxicity and easily accessible quencher that has advantages in practice. After measurement, the inner filter effect was corrected, and the Rayleigh and Raman scatterings were removed and interpolated. A median filter was further applied to the signal to remove the noise.

### 2.2 PARAFAC and Calculation of *F*_0_/*F*

PARAFAC was conducted with the Python package eempy^40^ using a hierarchical alternating least-squares (HALS) solver.^41^ With PARAFAC, EEMs were represented as weighted combinations of components. All EEMs shared the same components but with sample-specific weights. The weights were multiplied by the maximum excitation and emission loadings to obtain *F*_max_, which is the normalized component intensity indicator. EEMs of both original and quenched samples were used in the PARAFAC model establishment. The number of PARAFAC components was determined by calculating the average split-half similarity:

3 $$\overset{®}{\mathrm{sim}}=\overset{N}{\underset{i}{\sum }}\overset{R}{\underset{j}{\sum }}\left({\mathrm{sim}}_{\mathrm{ex}}+{\mathrm{sim}}_{\mathrm{em}}\right)/2$$

where sim_ex_ and sim_em_ are the similarity scores (Pearson correlation coefficients) in excitation and emission loadings between PARAFAC models established on two random splits of the EEM dataset. *R* denotes the number of components, and *N* denotes the number of split-half validations (*N* = 100 was used). The apparent quenching ratio *F*_0_/*F* of a PARAFAC component was calculated by dividing its *F*_max_ in the original sample by its *F*_max_ in the quenched sample according to eq 2.

### 2.3 Validation with Model Compounds

The purpose of studying model compounds is to understand the factors that can change the quantitative relationship between *F*_max_ and contaminant concentration, and how apparent *F*_0_/*F* reflects such change. Synthetic samples with highly similar tryptophan-like spectra but different compositions (Figure S1) were prepared by mixing bovine serum albumin (BSA) and *E. coli* at different ratios. Purified *E. coli* cells in 1/4 Ringer’s solution were prepared according to a previously reported protocol.^42^ BSA stock was prepared by dissolving BSA into 1/4 Ringer’s solution. *E. coli* at 0.7 million #/mL and BSA at 1.67 mg/L had similar fluorescence intensities and were used as baseline concentrations. Synthetic samples were prepared by mixing the baseline solutions in ratios of 0:1, 1:3, 1:1, 3:1, and 1:0. The synthetic sample EEMs were fitted with a one-component PARAFAC model to simulate component generalization. To quantitatively describe the relationship between *F*_max_ and *E. coli* or BSA concentrations, *E. coli*/*F*_max_ and BSA/*F*_max_ were calculated, which can be interpreted as the amount of *E. coli* or BSA concentration that one unit of *F*_max_ corresponds to. The evolution of apparent *F*_0_/*F*, *E. coli*/*F*_max_, and BSA/*F*_max_ in response to changing mixing ratios was investigated at different KI concentrations (0, 1.25, 2.5, 3.75, and 5 g/L). Another factor that can change the quantitative relationship between *F*_max_ and contaminant concentration is the concentration variations of intrinsic quenchers that exist in the original samples. To investigate this aspect, Suwannee River Humic Acid (HA) Standard II from the International Humic Substances Society (IHSS)^43^ was selected as the model intrinsic quencher. While fixing the KI concentration and the mixing ratio between *E. coli* and BSA, different concentrations of HA were spiked into the synthetic samples, and the changes in apparent *F*_0_/*F*, *E. coli*/*F*_max_, and BSA/*F*_max_ were investigated.

### 2.4 Validation with Real-World Greywater Samples

#### 2.4.1 Monitored System and Monitoring Targets

Distributed water reclamation systems have high demands on online monitoring.^44^ The uncertainties in influent quality, flow rate, and treatment efficiency lead to high variabilities in effluent concentrations and compositions (Table S1),^45,46^ providing a good validation platform for the effectiveness of apparent *F*_0_/*F*. In this light, an on-site greywater reclamation system consisting of a membrane bioreactor (MBR) and a biological granular activated carbon filter (BAC) was studied in July and October 2024 for validating apparent *F*_0_/*F* in water quality monitoring using EEM. The configuration and influent/effluent quality of the system are described in Text S1 and Table S1, respectively. To better cover the fluctuations in biological and chemical water quality under process uncertainties, samples were taken under various system conditions and sampling points, which are summarized in Table 1. In total, 130 samples were taken, stored at 4 °C, and analyzed in the laboratory within 24 h. The quenched EEMs and apparent *F*_0_/*F* were measured with a KI concentration of 2.5 g/L for all samples. Total cell count (TCC) and dissolved organic carbon (DOC) concentration were selected as predictive indicators for comprehensive monitoring of both microbial and chemical quality. Actual TCC was measured with a CytoFLEX flow cytometer (Beckman Coulter, USA) using SYBR Green I as the cell stain following a previously described protocol.^47^ Actual DOC was measured with a TOC analyzer (Shimadzu TOC-L, Japan) after filtration of the sample with a 0.45 μm filter. The performance of PARAFAC models built on different subsets of the samples is described in Table S2.

**Table 1:** Overview of the Operational Conditions and Sampling Points in the Sampling Campaign

Categories^a^	Name	Description	Number of samples in July	Number of samples in October
System conditions	Normal	Automated operation	25	44
	Low flow	Filtration stopped and water stagnated in BAC for at least 16 h before sampling	15	18
	High flow	Filtration was forced to be nonstop from at least 60 min before the sampling	0	18
	Simulated cross-connection	MBR effluent was added into the BAC effluent at volume ratios of 1/20 to 1/5.	0	10
Sampling points	BAC top	At ∼20% length of the filter bed	0	12
	BAC middle	At ∼50% length of the filter bed	0	13
	BAC bottom	At ∼80% length of the filter bed	0	12
	BAC effluent^b^	The BAC effluent pipe	40	53

a Samples taken under the same system conditions might come from different sampling points.

b Samples from “Simulated cross-connection” were counted as “BAC effluent”.

#### 2.4.2 EEM Self-Clustering by Apparent *F*_0_/*F*

It is found that including both unquenched and quenched samples in PARAFAC did not introduce significant bias in PARAFAC components compared to only including unquenched samples (Figure S3), ensuring the reliability of apparent *F*_0_/*F* in characterizing individual components specifically. Based on this, an EEM clustering approach (*F*_0_/*F*–K-PARAFACs) was developed to classify samples by apparent *F*_0_/*F*. The main goal of clustering was to observe whether in real-world samples the classification of apparent *F*_0_/*F* can automatically lead to the separation of samples with different quantitative relationships between *F*_max_ and TCC or DOC, and whether such differences are potentially linked to variations in DOM compositions due to treatment anomalies. If the clustering method can be validated, then it can also serve as a useful tool in unsupervised EEM analysis for chemical composition classification. *F*_0_/*F*–K-PARAFACs is adapted from the original K-PARAFACs with a different optimization objective to achieve the clustering of *F*_0_/*F*.^21^ The input is an EEM dataset, and the output is cluster labels for individual samples, with separate PARAFAC models established on each cluster. The method is described in detail in Text S2.

#### 2.4.3 Outlier Detection for New Sample in Real-Time Monitoring

In real-time water quality monitoring, a “training + testing” workflow is needed to provide exact numbers for contaminant concentration in new samples. First, a PARAFAC model was established using historical EEMs, and a linear relationship was fitted between *F*_max_ and TCC or DOC. For the contaminant quantification in an arriving new sample, the PARAFAC components were fitted to the new sample’s EEM to obtain *F*_max_, and TCC and DOC were predicted using the pre-established relationship. To detect abnormal samples and unreliable predictions in the testing phase, the distribution of apparent *F*_0_/*F* in training samples, after the removal of values with a *z*-score larger than 3, served as a reference for identifying outliers in new samples. If the apparent *F*_0_/*F* of a new sample fell outside the historical *F*_0_/*F* range, this new sample would be classified as an outlier. Other fluorescence indices and numerical error indicators, including the humification index (HIX),^48^ biological index (BIX),^49^ apparent quantum yield (AQY),^7^ reconstruction error, and relative reconstruction error^21^ were also calculated for comparison. The calculations of these indicators are described in Table S3. The outlier detection using these indicators followed the same protocol as apparent *F*_0_/*F* (i.e., filtering the samples with errors falling outside the reference range).

## 3 Results

### 3.1 Apparent *F*_0_/*F* vs Quantitative Relationship between Contaminant Concentration and *F*_max_: Association Verification with Model Compounds

Maintaining a stable quantitative relationship between the contaminant concentration and *F*_max_ is essential for using *F*_max_ as a reliable predictor. The model compound experiment was designed to demonstrate how this relationship can shift and how apparent *F*_0_/*F* serves as an indicator of such changes.

One obvious cause for the relationship shift is the change in the chemical compositions of compounds that contribute to the same *F*_max_. The use of *F*_0_/*F* utilizes the inherent variability of true *F*_0_/*F* across different compounds sharing the same fluorophore: If apparent *F*_0_/*F* shifts, it is possible that the share of compounds with different *F*_0_/*F* values has altered. For *E. coli* and BSA, it is observed that they exhibited significantly different apparent *F*_0_/*F* values at the same KI or HA concentration (Figure 1a,d). Compared to BSA, *E. coli* showed significantly less quenching in response to quenchers, potentially due to the protective role of cellular structures such as cell membranes or the embedding of fluorophores within shielding molecular complexes.^50^ Similar variations in quenching properties have also been reported in previous studies between other tryptophan-containing compounds, highlighting the diverse quenching properties of tryptophan fluorophores.^23,34,51^ Since apparent *F*_0_/*F* serves as a generalized indicator of the quenching ratio among fluorescent compounds with a shared fluorophore, its value may also reflect the predominant fluorescent compound. This is shown in Figure 1b,c, where the apparent *F*_0_/*F* decreased as *E. coli* contributed more to *F*_max_ (i.e., with a higher *E. coli* to BSA concentration ratio (E/B)), while the opposite trend was observed for BSA.

**Figure 1:**
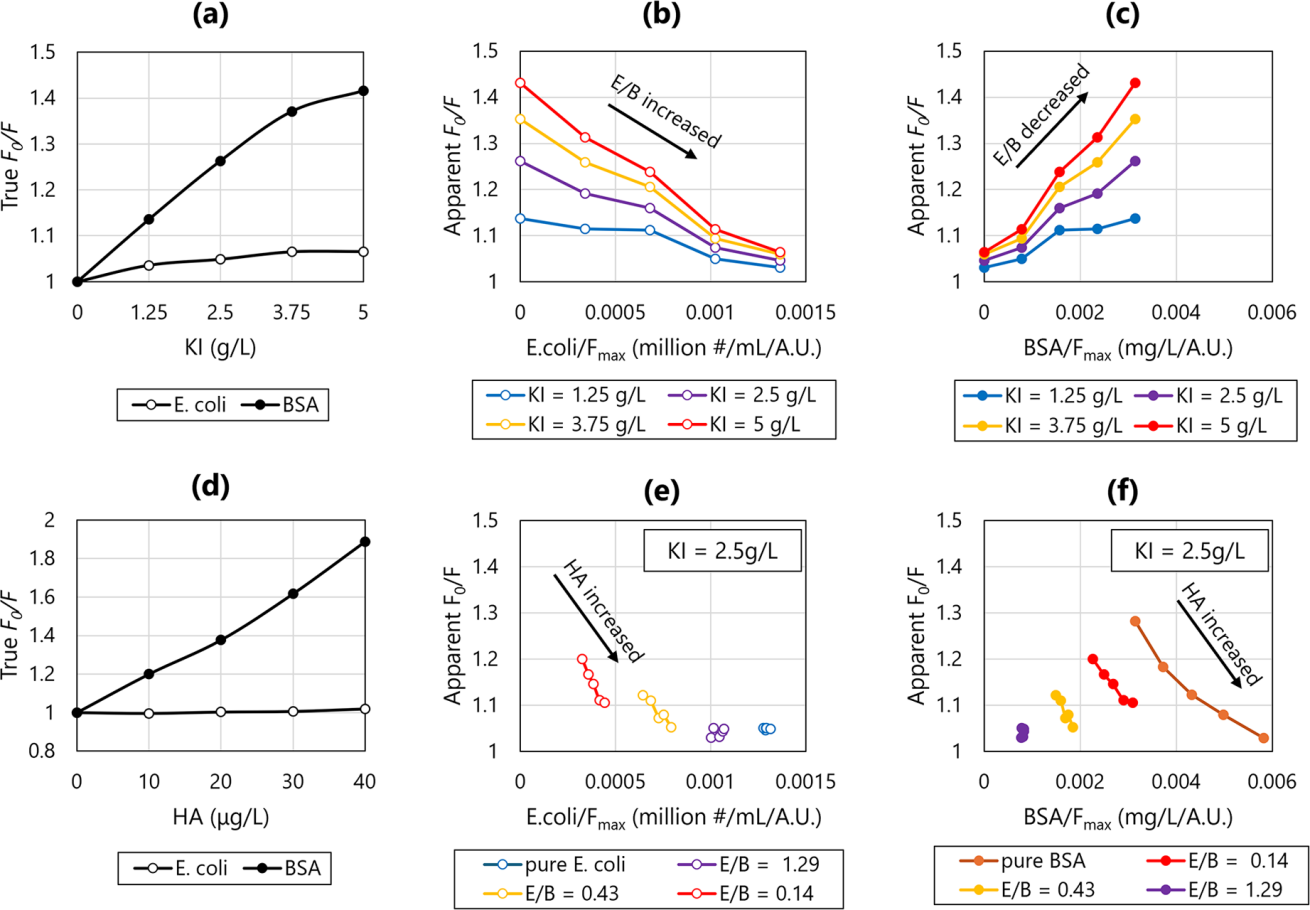
*F*_0_/*F* shift caused by (a–c) composition changes in compounds underlying the tryptophan fluorophore, and (d–f) variations in intrinsic quencher concentrations. (a,d) KI or HA concentrations versus true *F*_0_/*F* measured by the peak fluorescence of pure *E. coli* and BSA; (b,e) association between *E. coli*/*F*_max_ ratio and apparent *F*_0_/*F*; (c,f) association between BSA/*F*_max_ ratio and apparent *F*_0_/*F*. The key difference between (b,c) and (e,f) is that the variations in apparent *F*_0_/*F*, *E. coli*/*F*_max_, and BSA/*F*_max_ along each curve in (b,c) are caused by changing the mixing ratio between *E. coli* and BSA, i.e., E/B ratio with the unit (million #/mL)/(mg/L), while in (e,f) the variations along each curve are caused by changing the concentration of HA added to the sample before KI, without changing E/B and the KI concentration dosed (fixed at 2.5 g/L).

Another potential factor that can change the quantitative relationship between *F*_max_ and the monitored contaminant concentration is the variation in intrinsic quencher concentration. For example, Figure 1c shows that Suwannee humic acid (HA) is a strong quencher for BSA, which aligns with observations in previous studies.^23,34,52^ Figure 1e,f further illustrates that the change in HA concentration is a non-negligible factor for the apparent *F*_0_/*F* shift, which can occur even without changing the chemical composition of the compounds underlying a fluorophore. The impact of HA was observed to be more significant when BSA was the predominant fluorescent compound. For samples with a high share of *E. coli* and a low share of BSA, HA variations had only a little impact on both apparent *F*_0_/*F* and *E. coli*/*F*_max_. It is important to note that variations in HA concentration can offset the effects of the *E. coli*-to-BSA ratio (E/B), resulting in similar apparent *F*_0_/*F* values despite underlying differences in *E. coli*/*F*_max_ or BSA/*F*_max_. This compensatory effect is illustrated in Figure 1e,f, where curves corresponding to different E/B ratios exhibit overlapping apparent *F*_0_/*F* ranges. These findings suggest that while a shift in apparent *F*_0_/*F* reliably reflects changes in the quantitative relationship between *F*_max_ and contaminant concentrations, the inverse is not necessarily true—changes in *F*_max_-to-contaminant ratios may not always be reflected by shifts in apparent *F*_0_/*F*. Note that BSA might be more sensitive to HA quenching than other protein-like substances.^52^ The purpose of showing the interaction between BSA and HA is to cover even the “extreme case”.

### 3.2 Classification of Greywater Samples with *F*_0_/*F*–K-PARAFACs

Apparent *F*_0_/*F* must be further validated in real-world samples with more complicated chemical compositions. Therefore, *F*_0_/*F*–K–PARAFACs was implemented on all samples to distinguish samples by apparent *F*_0_/*F* levels—If apparent *F*_0_/*F* is an effective indicator, such differentiation in apparent *F*_0_/*F* should also lead to differentiation in the concentration-to-*F*_max_ ratio. The validation results are presented in Figure 2: as seen in Figure 2a, the clustering successfully separated samples into three groups with varying *F*_0_/*F* levels. Then, each of the four PARAFAC components (denoted as C1–C4) was associated with the relevant indicators. TCC was correlated with the tryptophan-like component C1, while DOC was correlated with other components that were mostly contributed by DOM. Upon comparison of the apparent *F*_0_/*F* of the four clusters, the largest and most consistent differences were observed between cluster 1 and cluster 4 across all components, with cluster 2 and cluster 3 generally representing intermediate states. Such differentiations were reflected in the nonoverlapping DOC/*F*_max_ between cluster 1 and cluster 4 in C3 and C4 (Figure 2b). In C1 and C2, although the TCC/*F*_max_ and DOC/*F*_max_ of clusters 2 and 3 did not strictly lie between clusters 1 and 4, the distinction between clusters 1 and 4 could still be clearly observed. However, it should be noted that the distributions of TCC/*F*_max_ and DOC/*F*_max_ within a single cluster might exhibit multiple peaks, indicating insufficient differentiation between varying contaminant concentration-to-*F*_max_ relationships. This observation was similar to the findings from the model compound experiments, where samples with differing concentration-to-*F*_max_ ratios yielded similar apparent *F*_0_/*F* values due to compensatory effects between shifts in fluorescent compound composition and intrinsic quencher concentration (Figure 1e,f).

**Figure 2:**
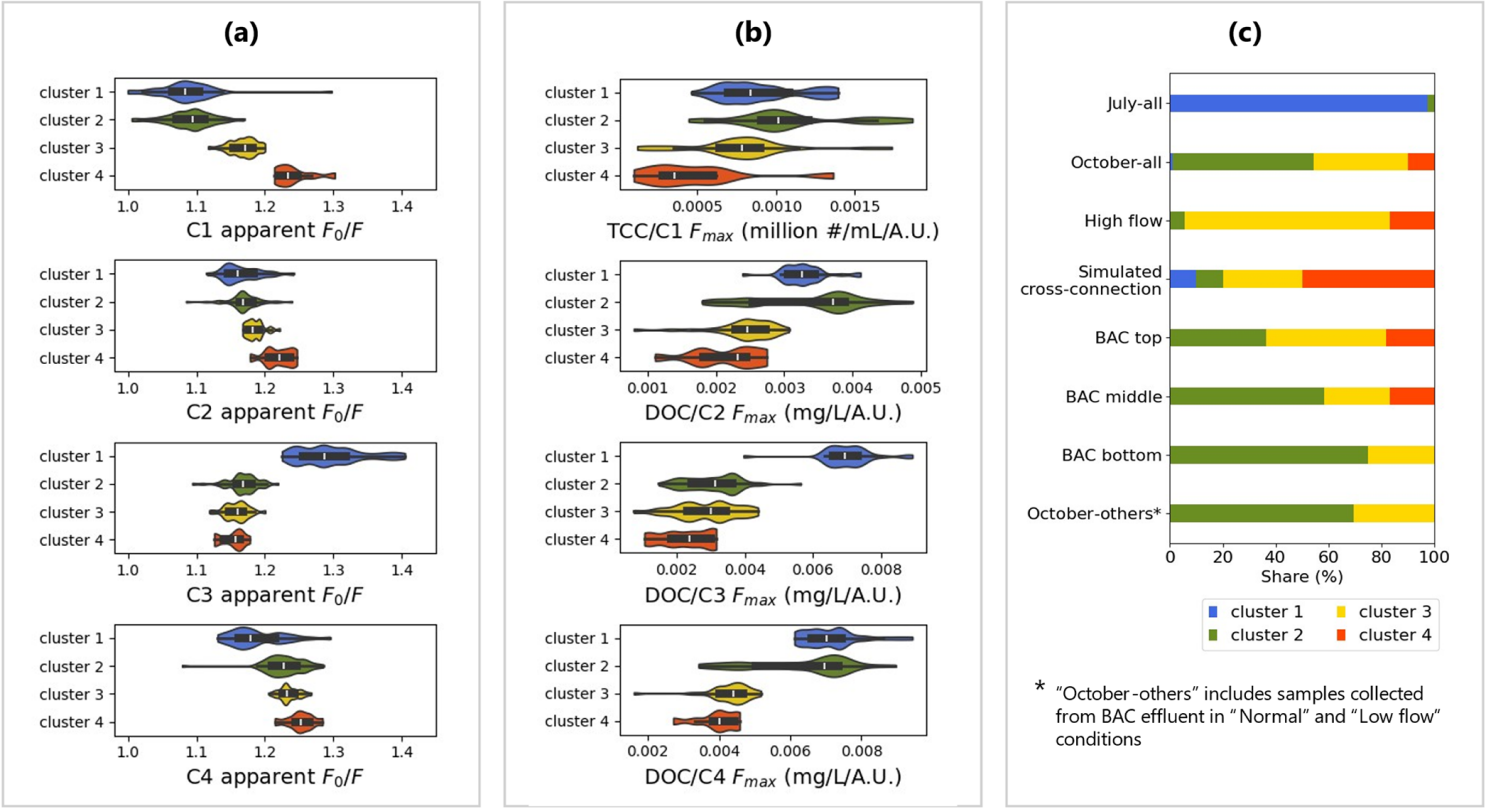
Distribution of apparent *F*_0_/*F*, TCC/*F*_max_, and DOC/*F*_max_ of clusters given by apparent-*F*_0_/*F*–K-PARAFACs. The clustering was applied to all EEMs (July + October samples). A KI concentration of 2.5 g/L was applied to all samples for the measurement of apparent *F*_0_/*F*. Note that the *F*_max_ and apparent *F*_0_/*F* of each cluster were calculated using the PARAFAC model built on samples within the cluster. (a) Violin plots of apparent *F*_0_/*F* of different components with PARAFAC established on individual clusters; (b) violin plots of *F*_max_/TCC or *F*_max_/DOC of different components with PARAFAC established on individual clusters; (c) shares of clusters in different sample categories.

Figure 2c further illustrates how samples from different times, system conditions, and sampling points were distributed across the clusters. Temporally, nearly all samples from July were assigned to cluster 1, with only one exception. Note that these samples from July were taken from BAC effluent in normal and low flow conditions, but their counterparts in October, which are labeled as “October-others” in Figure 2c, were assigned differently to other clusters. This suggests that the deviations in apparent *F*_0_/*F* observed in clusters 2 and 3 may be linked to long-term temporal variabilities in influent composition or system performance. For the October samples, both system conditions and sampling locations were found to influence cluster assignment. Samples collected under “High flow” or “Simulated cross-connection” conditions, as well as those taken from the top of the BAC column, were more frequently classified into cluster 3 or cluster 4. It can be speculated that there might be two factors driving the distinction between cluster 2 and cluster 3—the insufficient treatment of DOM from MBR effluent and the DOM produced by biological activities enriched in the upper part of the BAC column. This result implies the potential of apparent *F*_0_/*F* in identifying system anomalies by reflecting the chemical composition changes underlying the fluorophores.

### 3.3 Physical Interpretability and Robustness of Apparent *F*_0_/*F* in Greywater Samples

With the clustering analysis, the statistical relevance of the apparent *F*_0_/*F* shift in indicating changes in the quantitative relationship between *F*_max_ and contaminant concentration has been demonstrated. Beyond the occurrence of an apparent *F*_0_/*F* shift itself, the direction and degree of the shift might also carry physically meaningful information about the change in fluorescent compound compositions. According to the previous analysis on *E. coli* and BSA mixtures, the lower the apparent *F*_0_/*F*, the higher the share of *E. coli*. Based on this, a generalized hypothesis was made: in greywater, the bacteria had similarly low quenching sensitivity as *E. coli* due to cellular protection, while the DOM with tryptophan fluorophore had higher sensitivity. If apparent *F*_max_ shifts toward a larger number, then it suggests the contribution of bacterial fluorescence to *F*_max_ decreases. To validate this hypothesis, we studied the apparent *F*_0_/*F* shift in tryptophan-like component C1. The methodology is described in Figure 3a using the full sample set as an example (the clustering output was identical to that in Figure 2a). It is found that after removing cluster 3 with outlying apparent *F*_0_/*F*, the remaining samples exhibited a better correlation with TCC. To verify the robustness of this result, random datasets were generated and tested with the outlier sample removal method (Figure 3b,c). Depending on the selection of samples, the correlation between C1 *F*_max_ and TCC differed: with only samples from October, it was not even possible to establish statistically significant (*p* < 0.05) correlations. Nevertheless, the correlation coefficients were increased to different degrees, and particularly for datasets with only October samples, 90% of the random datasets became statistically significant. The success in the robustness test provides supporting evidence for the physical interpretability of apparent *F*_0_/*F*, as it demonstrates the coherence of apparent *F*_0_/*F* with the actual *F*_0_/*F* even under the numerical uncertainties (e.g., component shape change and measurement noise variations) introduced by random sample combinations.

**Figure 3:**
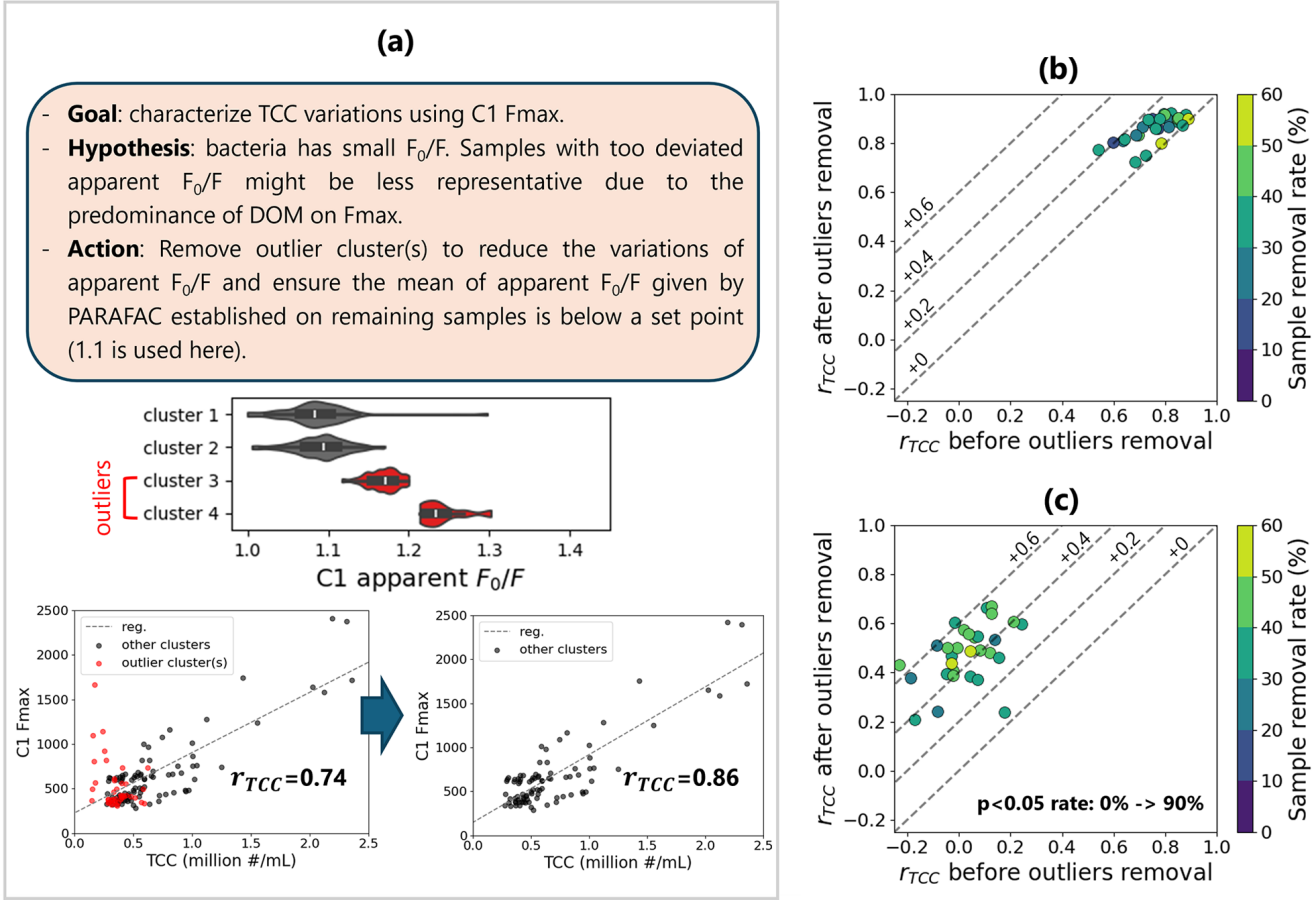
Using *F*_0_/*F* to identify outlier samples with *F*_max_ uncorrelated to TCC. (a) An overview of the method using the whole sample set (July + October) as an example. The Pearson correlation coefficient between C1 *F*_max_ and TCC (*r*_TCC_) was improved after outlier removal; (b,c) tests on the method robustness, which were performed by repeatedly applying the same method on different random sample sets of size 30 with (b) 30% of samples from July and 70% of samples from October, and (c) 100% of samples from October. With only October samples, correlations with *p* < 0.05 could not be established before outlier removal. After outlier removal, 90% of the random datasets exhibited *p* < 0.05 correlations between C1 *F*_max_ and TCC.

### 3.4 Apparent *F*_0_/*F* for Model Failure and System Anomaly Detection in Real-Time Monitoring

As the clustering method has proven its potential in filtering outliers with the assistance of prior knowledge of true *F*_0_/*F*, an outlier removal strategy adapted for real-time monitoring could be outlined: If a contaminant quantification model with good training accuracy is established using historical samples, then the apparent *F*_0_/*F* values of those samples can serve as a reference. When a new sample exhibits an apparent *F*_0_/*F* that falls outside the typical range observed in the historical dataset, it can be flagged as an outlier since the model might make unreliable predictions for it due to potential inconsistencies in chemical composition between the historical samples and the new sample. To validate this approach, we identified that samples in July were more suitable for model establishment due to higher correlations (Table S2). We trained TCC and DOC prediction models using the samples in July and applied the models to predict TCC and DOC samples in October. The outlier detection method mentioned above was implemented, and the results of the prediction accuracies and outlier identifications are plotted in Figure 4. As shown in Figure 4a–c, the distribution of *F*_0_/*F* in C1, C2, and C3 exhibited varying degrees of shift in the test phase. Depending on the extent of this shift, different proportions of October samples were identified as outliers (C1: 47%, C2: 21%, C3: 100%). The relationships between TCC or DOC and *F*_max_, along with the relative prediction errors in training and testing, are shown in Figure 4d–i, where the identified outlier samples exhibited significantly higher relative errors compared to both the training samples and the nonoutlier test samples. This aligns with findings in Figure 2 that apparent *F*_0_/*F* is associated with TCC/*F*_max_ or DOC/*F*_max_, since relative error is substantially a metric to quantify the shift in TCC/*F*_max_ or DOC/*F*_max_ from training samples to testing samples. Notably, changes in DOC/*F*_max_ in a single component could be propagated to changes in relationships between DOC and multicomponent indicators. For instance, multivariate models using multiple *F*_max_ for DOC prediction also exhibited significant error (Figure S4), indicating that apparent *F*_0_/*F* is not only an indicator for single-*F*_max_ models but also reflects the predictability of multivariate models that eventually also rely on the robustness of single *F*_max_. Another attempt is to calculate apparent *F*_0_/*F* using peak-picked fluorescence intensities instead of PARAFAC-derived *F*_max_ (Figure S5). However, this approach was less effective in identifying high-error outliers, highlighting the importance of PARAFAC in isolating the fluorophore-specific signal for more meaningful calculation of apparent *F*_0_/*F*.

**Figure 4:**
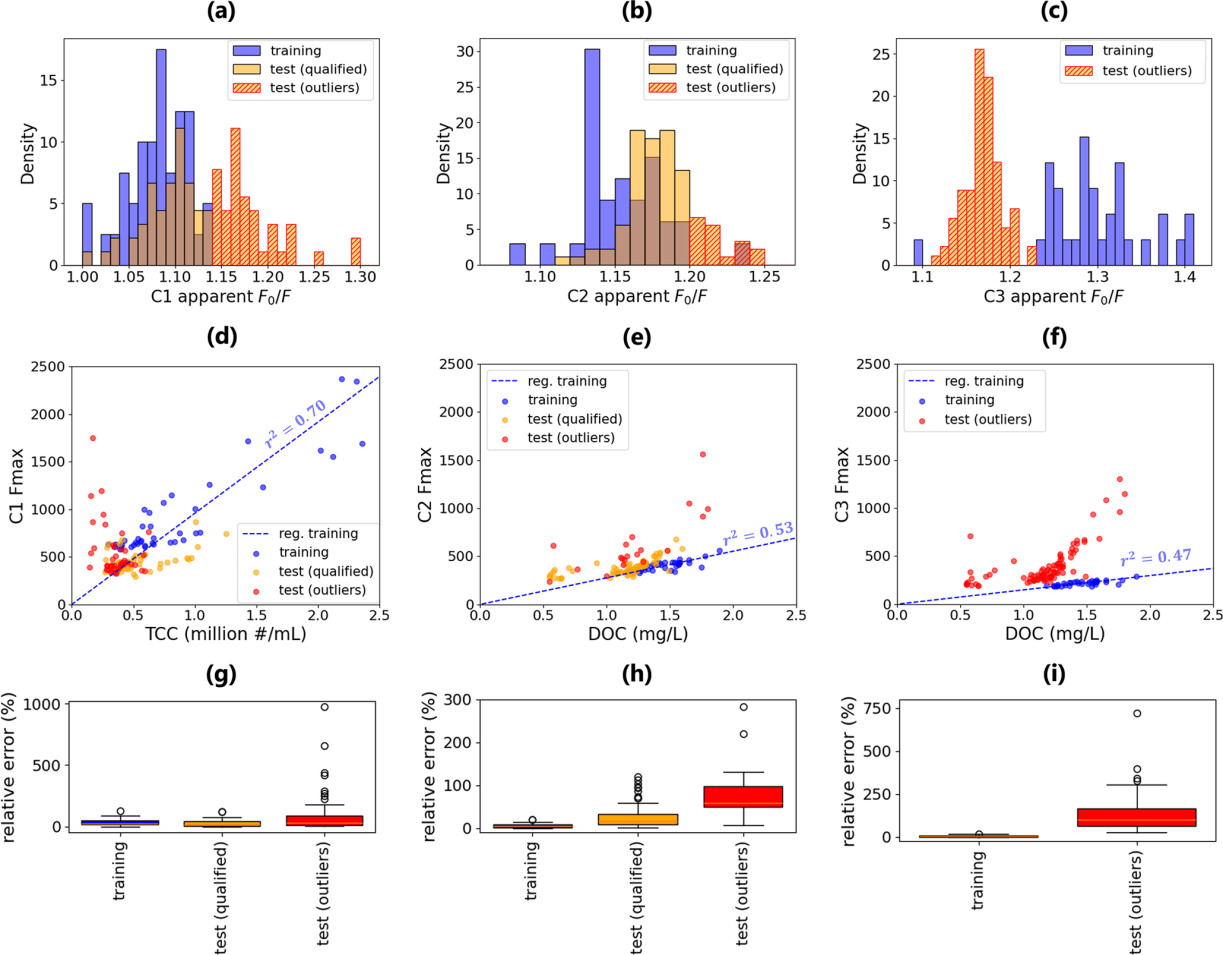
Performance of the apparent *F*_0_/*F* in identifying outliers for real-time monitoring. A four-component PARAFAC model was trained on samples from July. TCC and DOC prediction models were developed using the component with the highest Pearson correlation coefficient (C2 and C3 showed similar performance, so both are displayed). The models were then independently tested on individual samples from October. (a–c) Density histograms of *F*_0_/*F* of (a) C1, (b) C2, and (c) C3 in training and test; (d–f) relationships between the predicted analytes and *F*_max_ for (d) C1, (e) C2, and (f) C3 in training and test. The *r* ^2^ values displayed are calculated from training; (g–i) relative error (the ratio of the absolute error to the true value) of prediction using (g) C1, (h) C2, or (i) C3 in training and test.

Figure 5 compares the performance of apparent *F*_0_/*F*, conventional fluorescence indices, and numerical error metrics in detecting high predictive errors (Figure 5a) and system anomalies (Figure 5b). First, using C3 to predict DOC shall be highlighted as a special case, as 92.2% of the predictions in the test phase had relative errors higher than 50%. According to the PARAFAC model database OpenFluor,^53^ there are no matches with high similarity (Tucker’s congruence >0.98 in both excitation and emission loadings) with reported models in C3, while in all other components, there are at least 5 matches, including models established on samples from full-scale centralized water recycling plants.^54^ Given that no PARAFAC models on OpenFluor have been developed specifically for greywater samples (as of June 2025), the absence of matching components for C3 may suggest that it originates from chemical compounds unique to greywater influent. The compounds contributing to C3 might undergo a composition change, possibly due to high influent composition variabilities in a small-scale system. Such a composition shift was strongly indicated by apparent *F*_0_/*F*, HIX, and relative RE with a 100% outlier rate. However, for TCC predictions made by C1 *F*_max_ and DOC predictions made by C2 *F*_max_, none of the indicators achieved very ideal outlier detection performance, i.e., consistently identifying all large-relative-error predictions while avoiding false positives among small-relative-error predictions (Figure 5a). Despite such limitations, apparent *F*_0_/*F* stood out as the only indicator that demonstrated a consistent increase in outlier rates from small-relative-error to large-relative-error categories for both C1 and C2, showing a >50% difference in outlier rates between the 0–25% and >100% error categories. Although other indicators (e.g., RE for C1 and AQY_320_ for C2) showed some similar trends for a single component, their maximum outlier rate differences were smaller (<30%) and lacked consistent performance across different components. Most large-relative-error samples that were not identified by apparent *F*_0_/*F* concentrations occurred at low TCC and DOC concentration levels. When samples were instead categorized by absolute error, apparent *F*_0_/*F* exhibited even greater outlier rates for large-absolute-error predictions (Figure S7), despite being hypothesized as more associated with relative error. Furthermore, the ability of each indicator to detect system anomalies was also evaluated (Figure 5b). Apparent *F*_0_/*F* for C1 and C2, along with AQY_254_ and BIX, was able to exhibit higher outlier rates in “abnormal” scenarios not present during the training phase, such as “High Flow”, “Simulated cross-connection”, and BAC shortcuts (i.e., sampling directly at the BAC column). Compared to AQY_254_ and BIX, apparent *F*_0_/*F* presented more moderate outlier rates while also reflecting different anomaly types: C1 apparent *F*_0_/*F* was responsive to system condition changes from “Normal” and “Low flow” to “High flow” and “Simulated cross-connections”, whereas C2 apparent *F*_0_/*F* showed higher sensitivity to the sampling location. These results demonstrate the different outlier patterns between PARAFAC components and highlight the unique capability of apparent *F*_0_/*F* in providing component-specific outlier information, in terms of both relative errors and treatment anomalies.

**Figure 5:**
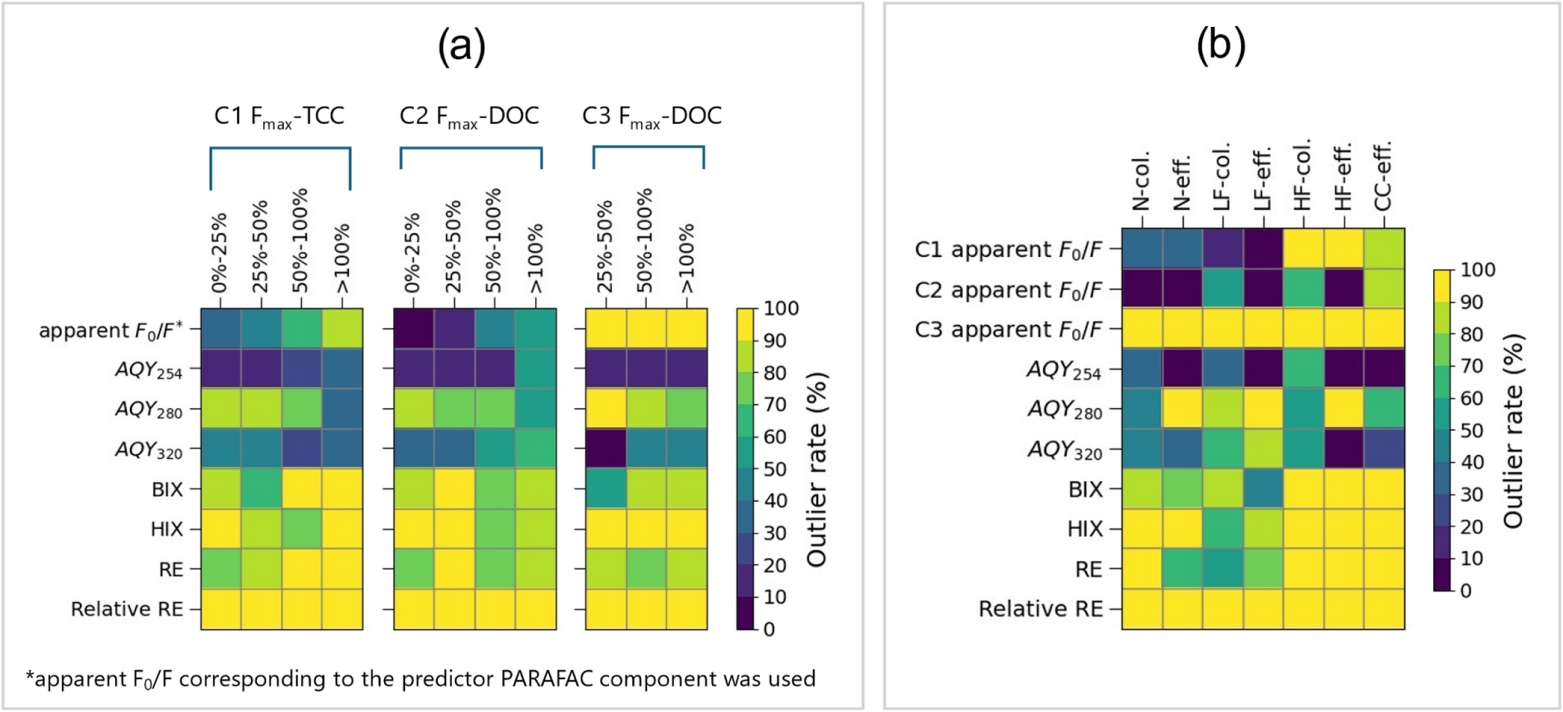
Outlier rates in model testing are given by different indicators (vertical axis) in different sample categories (horizontal axis). (a) Samples are categorized by relative errors. Predictions of TCC or DOC made by C1, C2, and C3 were plotted on the left, middle, and right, respectively. All DOC predictions using C3 have relative errors above 25%. (b) Samples are categorized by system conditions and sampling locations. N: “Normal”; LF: “Low flow”; HF: “High flow”; CC: “Simulated cross-connection”; col.: column samples; eff.: effluent samples.

## 4 Discussion

### 4.1 Mechanistic Basis for the Effectiveness of Apparent *F*_0_/*F*

With the results of model compound analysis, two factors that may simultaneously change apparent *F*_0_/*F* and the contaminant concentration-to-*F*_max_ ratio have been revealed. One is the composition of fluorescent compounds contributing to the same *F*_max_, and the second is the variation in intrinsic quencher concentration that affects the fluorophore. For the former factor, the mechanistic explanation is relatively straightforward: the compounds underlying the fluorophore naturally have different *F*_0_/*F*. When they are mixed at different ratios, their shares of contributions to *F*_max_ would change, resulting in a shifted quantitative relationship between *F*_max_ and concentrations, as well as different apparent *F*_0_/*F*. For the latter, the influence of intrinsic quencher concentration on *F*_max_ per unit of contaminant concentration is certain, but why apparent *F*_0_/*F* also changes with the intrinsic quencher concentration needs to be explained—essentially, this implies that the apparent *F*_0_/*F* is a function of not just extrinsic quencher concentration (*Q*_e_) but also intrinsic quencher concentration (*Q*_i_). The derivation of apparent *F*_0_/*F* with the presence of both quenchers can be conceptualized as follows:^39,55^

4 $$F_{0,\mathrm{true}}/F_{0,\mathrm{obs}}=f\left(K_{i},Q_{i}\right)$$

5 $$F_{0,\mathrm{true}}/F=g\left(K_{i},K_{e},Q_{i},Q_{e}\right)$$

6 $$\mathrm{apparent}\;F_{0}/F=F_{0,\mathrm{obs}}/F=\frac{F_{0,\mathrm{true}}/F}{F_{0,\mathrm{true}}/F_{0,\mathrm{obs}}}=\frac{g\left(K_{i},K_{e},Q_{i},Q_{e}\right)}{f\left(K_{i},Q_{i}\right)}$$

where *F*_0,true_ refers to the theoretical fluorescence intensity without any quenchers. *F*_0,obs_ refers to the *F*_max_ in the presence of an intrinsic quencher, which is also the measured *F*_max_ before adding the quencher. *F* refers to the measured *F*_max_ after adding the extrinsic quencher. *K*_i_ and *K*_*e*_ refer to the quenching parameters for intrinsic and extrinsic quenchers, respectively. Therefore, the key is to ensure Q_i_ not being eliminated in eq 6. Such elimination would happen if the quenching from intrinsic and extrinsic quenchers is completely “multiplicative”:

7 $$F_{0,\mathrm{true}}/F_{0,\mathrm{obs}}=1+K_{i}\left[Q_{i}\right]$$

8 $$F_{0,\mathrm{true}}/F=\left(1+K_{i}\left[Q_{i}\right]\right)\left(1+K_{e}\left[Q_{e}\right]\right)$$

9 $$\mathrm{apparent}\;F_{0}/F=1+K_{e}\left[Q_{e}\right]$$

In the derivation of eqs 7–9, it is assumed that the effects of each quencher operate independently without influencing each other’s binding or dynamic behavior. Although previous studies suggest that iodide typically causes dynamic quenching^56^ while HA exhibits predominant static quenching to proteins,^34^ our results do not support the independence between KI quenching and HA quenching, as higher concentrations of HA were observed to weaken KI’s quenching effect (Figure 1d,e). One speculation is that when HA binds to BSA, it may shield the fluorophores through hydrophobic domains or cause conformational alterations in BSA, reducing the accessibility of fluorophores to KI.^33,57^ The model compound experiments are designed to showcase the causal relationship between shifts in apparent *F*_0_/*F* and the loss of *F*_max_ predictability, as well as provide prior knowledge about the quenching behaviors of bacteria, protein, and humic acids. While Suwannee River HA may not be representative of greywater DOM, it was selected due to its well-characterized static quenching behavior, which allowed us to test how distinct quenching mechanisms interact. These experiments are not intended to reproduce the exact behavior of greywater DOM, but rather to support the theoretical basis of the apparent *F*_0_/*F* approach. A comprehensive understanding of the quenching dynamics of DOM in wastewater systems remains an important direction for future research.

Another mechanistic aspect is the relationship between the apparent *F*_0_/*F* value and the composition of the fluorescent compounds. As a generalized indicator, apparent *F*_0_/*F* combines the true *F*_0_/*F* values of various compounds sharing a similar fluorophore basis, weighted by their contributions to fluorescence intensity. This is coherently validated from synthetic samples to greywater samples: due to the low quenching sensitivity of bacteria, samples with higher *F*_max_ in the tryptophan-like component consisted of more nonbacterial fluorescence (Figure 1), resulting in *F*_max_’s low representativeness for TCC (Figure 3).

### 4.2 Applicability and Limitations of Apparent *F*_0_/*F* in Water Quality Monitoring

In this study, the applications of apparent *F*_0_/*F* in outlier detection are showcased with the monitoring of TCC and DOC in a greywater reclamation system. First, a self-clustering approach with no need for training was developed to classify samples. The goal of this method is to separate samples exhibiting different quantitative relationships between *F*_max_ and the concentrations of underlying fluorescent compounds—differences that may be associated with anomalies in the treatment process or influent quality. If prior knowledge of contaminant-specific *F*_0_/*F* is available, it is possible to further interpret the exact composition changes and exclude samples with uncorrelated *F*_max_ from the contaminant quantification (Figure 3). Therefore, the clustering method is useful in analyzing precollected environmental samples, enabling the identification of shifts in chemical composition (e.g., changing shares of fluorescent compounds or intrinsic quencher concentrations), and potentially providing a more reliable quantitative characterization of fluorescent compound dynamics.

For real-time monitoring, a thresholding method is proposed to identify individual new samples with apparent *F*_0_/*F* falling outside the normal range given by the historical samples. This method provides valuable information about the changes in concentration-to-*F*_max_ relationships that lead to model failures and identifies potential system anomalies. Although other fluorescence indices and numerical error metrics are available, they are not able to provide component-specific or fluorophore-specific information. In contrast, apparent *F*_0_/*F* can reveal the composition and photophysical changes that are hidden behind components that appear to be spectrally stable. With recent developments in hardware, online EEM measurement is possible using an autosampler.^21,58,59^ The automatic measurement of apparent *F*_0_/*F* would require only an additional quencher dosing device, enabling improved model failure and system anomaly detection in real time. Moreover, apparent *F*_0_/*F* has the potential to serve as a diagnostic tool to inform the need for recalibration, supporting the development of a more adaptive and robust monitoring framework.

Nevertheless, it is necessary to understand the limitations of apparent *F*_0_/*F*. First, as results in model compound samples (Figure 1e,f) and greywater samples (C2 in Figure 2) suggest, there is a certain risk that the loss in *F*_max_’s predictability is not reflected on apparent *F*_0_/*F* if multiple mechanisms (e.g., concentration changes involving multiple intrinsic quenchers or spectrally similar compounds) counterbalance each other’s impacts, leading to insufficient outlier capture rates for high-error samples (Figure 5a). To mitigate this risk, one possible strategy is to introduce multiple quenchers with distinct mechanisms or apply different quencher concentrations. By doing so, additional variation in apparent *F*_0_/*F* may be elicited, and samples exhibiting similar apparent *F*_0_/*F* under one quenching condition may show distinguishable differences under another,^37,60^ thus enhancing diagnostic capability. The use of KI in this study primarily targets solvent-accessible, hydrophilic regions of DOM. Future studies are recommended to investigate quenchers that access more hydrophobic domains or operate via different mechanisms, such as dynamic vs static quenching, to capture a broader range of DOM interactions and improve compositional resolution. Another possibility is to conduct high-frequency EEM measurements and determine outliers based on outlier rates among multiple samples within a specific time window. Moreover, the generalization of apparent *F*_0_/*F* for trace contaminant monitoring should be done with caution. Unlike bacteria or general DOM that directly affects apparent *F*_0_/*F* through their contribution to *F*_max_, trace contaminants below μg/L concentration levels have no direct impact on *F*_max_ and apparent *F*_0_/*F*. Therefore, attempts at quantifying trace contaminants rely on other fluorescent compounds as proxies. With apparent *F*_0_/*F*, what is possible is to determine whether *F*_max_ represents the same proxy compound composition, but it does not guarantee the mechanistic association (e.g., relevance in removal behavior in treatment) between the proxies and the trace contaminant. Therefore, having consistent apparent *F*_0_/*F* does not automatically ensure the effectiveness of trace contaminant quantification models—the mechanistic relevance of the proxy fluorescent compounds must also be validated.

### 4.3 Environmental Implications

This work underscores both the need and the potential for EEM analysis at the subcomponent or subfluorophore-group level. The need arises from limitations in existing EEM analysis tools, which fall into three main categories: The first includes black-box data-driven methods. Although efforts in interpreting the model have been made, the lack of physical coherence (e.g., “high importance” pixel distributions were scattered or conflicted with physical knowledge) raises concerns about overfitting and limits their practical utility.^11,12,61,62^ The second category includes semi-interpretable methods at the bulk level, such as peak-picking, regional integration, HIX, BIX, and AQY. These tools are grounded in broad domain knowledge, such as the representative EEM region of different types of fluorescent compounds,^63,64^ or the physical-chemical properties such as quantum yield. However, they often neglect signal overlap from chemically independent compounds, leading to significant interpretation ambiguity when these tools are applied for composition characterization^26,65^ or outlier detection as shown in this work. The third category includes semi-interpretable methods at the component or fluorophore-group level, including PARAFAC *F*_max_ of individual components and *F*_max_ ratios between PARAFAC components.^66^ While good local correlations were demonstrated in many studies, the robustness of such methods might still be vulnerable to compositional and photophysical changes within fluorescent compounds contributing to the same PARAFAC component. The quenching approach is a valuable primary demonstration of how subcomponent or subfluorophore-group level information can be extracted and used to address the critical limitations of existing methods in practical water quality monitoring. We recommend that future fluorescence-based monitoring research explores the following directions:

Moderating quenching agents or other perturbations as potential new dimensions apart from excitation and emission wavelengths for designing fluorescence sensing techniques. To unlock better interpretation of perturbation-derived indicators like apparent *F*_0_/*F*, their relationships with DOM physicochemical properties (e.g., hydrophobicity, aromaticity) should be investigated using real-world samples.
Utilize the perturbations to develop new numerical constraints for EEM processing. So far, PARAFAC’s “linearity assumption” that the emission spectrum shape is consistent regardless of excitation wavelength is the only physically based numerical constraint that has been successful. As questions remain about the accuracy and sufficiency of this assumption, perturbation-based strategies may enable the introduction of new constraints. For example, regulating the variations in apparent *F*_0_/*F* for individual components may help achieve more physically interpretable signal decomposition, as in ideal situations, a component should have a constant *F*_0_/*F* if it represents the same fluorescent compounds across all samples.

## Supplementary Material



## Notes

Greywater description; details of *F*_0_/*F*–KPARAFACs; complementary analysis for real-time greywater monitoring (PDF)
