# Supplementary material for: Fluorescence Quenching as a Diagnostic Tool for Prediction Reliability Assessment and Anomaly Detection in EEM-Based Water Quality Monitoring
Source: Environ Sci Technol. 2025 Sep 5;59(36):19490–501. doi: 10.1021/acs.est.5c05952 (PMC12444986; doi:10.1021/acs.est.5c05952)
Supplement: Supplementary file 1 [file es5c05952_si_001.pdf]

Supporting Information for

Fluorescence Quenching as a Diagnostic Tool for Prediction Reliability Assessment and Anomaly Detection in EEM-Based Water Quality Monitoring

Yongmin Hu [a, b], Céline Jacquin [a, c], Eberhard Morgenroth [a, b, *]

[a] Eawag: Swiss Federal Institute of Aquatic Science and Technology, 8600 Dübendorf, Switzerland

[b] ETH Zürich, Institute of Environmental Engineering, 8093 Zürich, Switzerland

[c] Gujer AG, 8153 Rümlang, Switzerland

* Corresponding author: Eberhard Morgenroth (Eberhard.Morgenroth@eawag.ch)

Number of pages: 15

2 Text Sections

7 Figures

3 Tables

TABLE OF CONTENTS

Text S1. Description of the greywater reclamation system 3

Text S2. Description of F_0/F-K-PARAFACs clustering...4

Figure S1. EEMs of *E. coli* and BSA. 6

Figure S2. The scheme of the F_0/F-K-PARAFACs clustering algorithm.......................................7

Figure S3. Excitation and emission loadings of PARAFAC models built on different dataset.......8

Figure S4. Training and testing of multivariate linear regression models using Fmax of multiple components...9

Figure S5. Performance of apparent F_0/F calculated from fluorescence intensities through peak-picking. 10

Figure S6. Density histograms of different fluorescence indices and error metrics.. 11

Figure S7. Outlier rates in model test given by different indicators (vertical axis) in absolute error categories.. 12

Table S1. Influent and effluent quality (25th, 50th and 75th quantiles) of the greywater reclamation system.. 13

Table S2. Overview of PARAFAC models trained on different timescales using both unquenched and quenched samples.. 13

Table S3. Description of different fluorescence indices and EEM error metrics..........................14

References 15

Text S1. Description of the greywater reclamation system

The treatment consisted of a membrane bioreactor (MBR) and a granular activated carbon (BAC) filter. The MBR tank had a volume of 1600 L with an average inflow of 730 L/d and a hydraulic retention time of 1.5-3 d. A water level threshold was set, and once the water level reached the threshold, the water would be filtered with 6 AQUALOOP ultrafiltration membrane modules (INTEWA, Germany). The membranes were made of Polyethylene (PE) and had an average pore size of 0.02 µm and a total surface area of 6 m^2. The BAC filter was filled with granular activated carbon (Chemviron F-400) and had an empty bed contact time (EBCT) of 112 minutes. Due to the intermittency of greywater inflow, the activation of MBR filtration was also intermittent in normal automated conditions.

Text S2. Description of F_0/F-K-PARAFACs clustering

Figure S1 provides a scheme of the clustering method. In general, the method is mostly the same as the K-PARAFACs introduced in our previous work. [1] The differences are highlighted in bold:

1) Parameter selection: Determine K (the number of clusters) and R (the number of components for each PARAFAC model established on individual cluster). The optimal value for K is identified based on the value that yields the highest Silhouette score of the consensus matrix, which is introduced in step 7 and 8. The optimal R is chosen according to the optimal number of components for the unified PARAFAC model based on variance explained and core consistency (Bro and Kiers, 2003).

2) Sub-sampling: Randomly select 80% of the EEMs in the original EEM dataset. Steps 3 to 6 will only apply to the selected EEMs.

3) Random labeling: Randomly split the EEM dataset into K sub-datasets and label them from 1 to K.

4) PARAFAC models establishment: Given the labels of EEMs, construct an R-component PARAFAC model for each of the sub-datasets where the number of EEMs is greater than R. If all sub-datasets have enough EEMs, in total, there will be K PARAFAC models from K sub-datasets. **Calculate the mean values of apparent F_0/F in all components for each model. These mean values are used as "centroids" for each model.**

5) Re-labeling of each EEM: Given the K PARAFAC models, reconstruct each EEM using the components of each of the K PARAFAC models by linear regression without intercept. **Afterwards, calculate the apparent F_0/F of individual samples, the EEM is relabeled according to the closest apparent F_0/F centroids.**

6) Check for convergence: **Iterate steps 4 and 5 until no significant change (overall Tucker's congruence > 0.998) can be observed in cluster-specific PARAFAC models between two iterations.** Then, end the algorithm and use the current labels and corresponding PARAFAC models as the final output.

7) Evaluate consensus: Repeat steps 2 to 6 (i.e., base clustering) N times to obtain N outputs. Note that the output labels of each time of base clustering might differ due to the randomness of steps 2 and 3. Based on the labels of the N outputs, calculate the consensus matrix, in which the similarity between a pair of EEMs is represented by the consensus coefficient (i.e., the ratio of the number of base clusterings in which the two EEMs were grouped to the total number of base clusterings in which both EEMs were not discarded in the sub-sampling step).

8) Hierarchical clustering: Subtract the consensus coefficients from 1 to obtain the distance coefficient between each pair of samples (the larger the distance, the less similar the two samples are). Conduct hierarchical clustering with the distance coefficients and obtain the final clustering output. The optimal number of clusters was determined by the Silhouette score. By sorting the EEMs according to the final clustering output, the consensus matrix was reformulated in a way that blocks of high consensus coefficients appeared along the diagonal of the consensus matrix – Each block represents one cluster. **If a cluster has samples less than the number of PARAFAC components +1, combine this cluster to the closest cluster.**

Supporting Figures

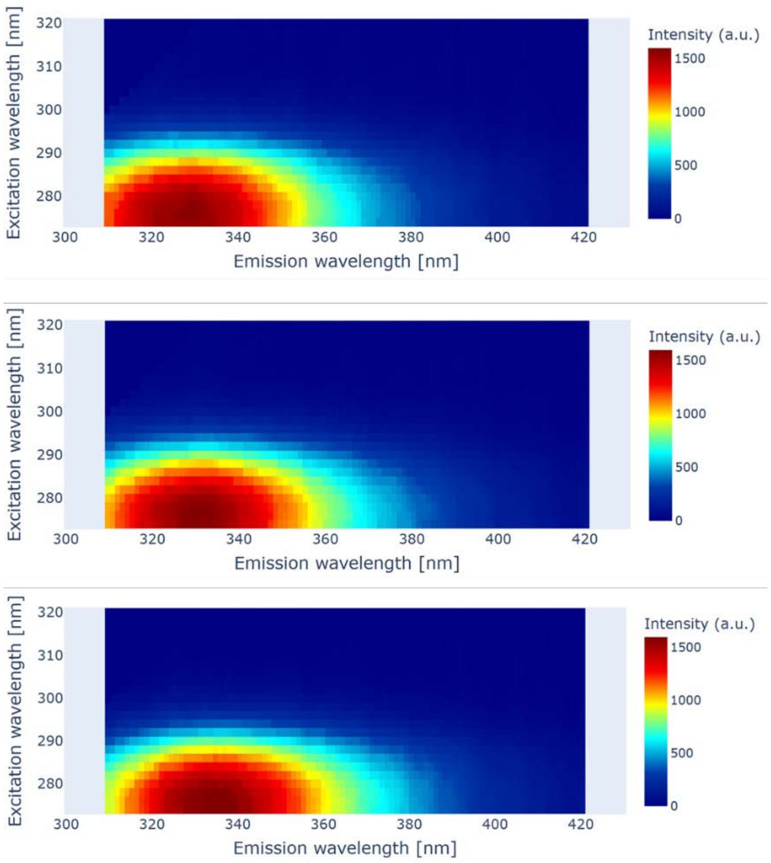

Figure S1. EEMs of (top) pure E. coli, (middle) 0.35 million #/mL of E. coli with 0.83 mg/L BSA, and (bottom) pure BSA.

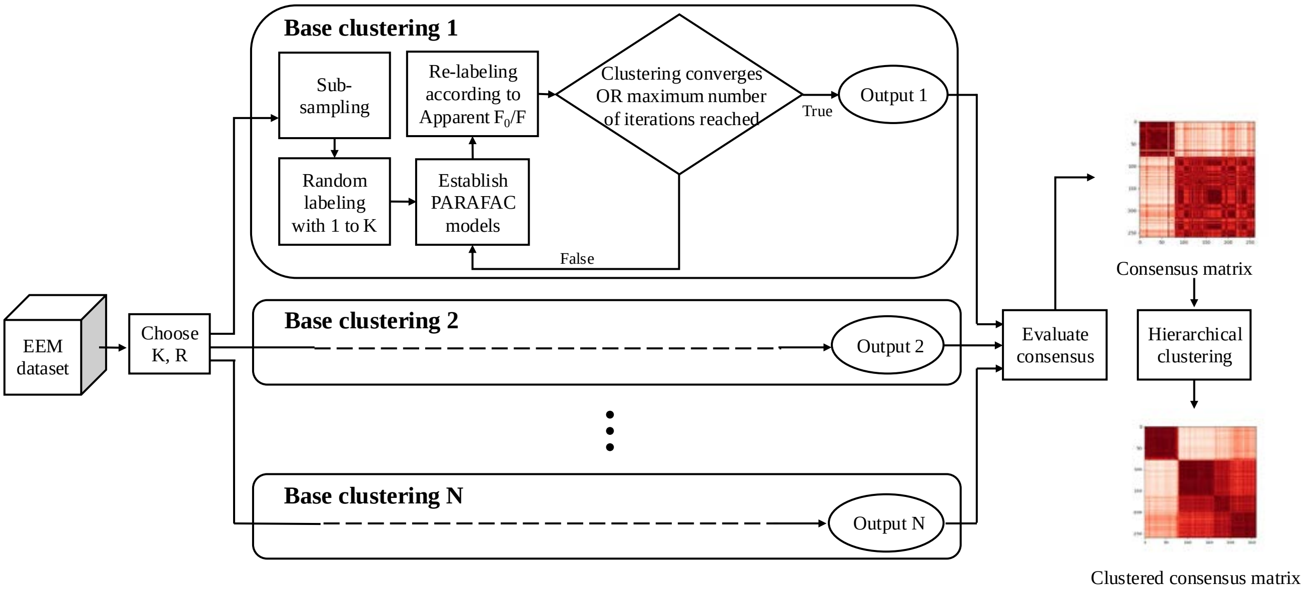

Figure S2. The scheme of the F_0/F-K-PARAFACs clustering algorithm.

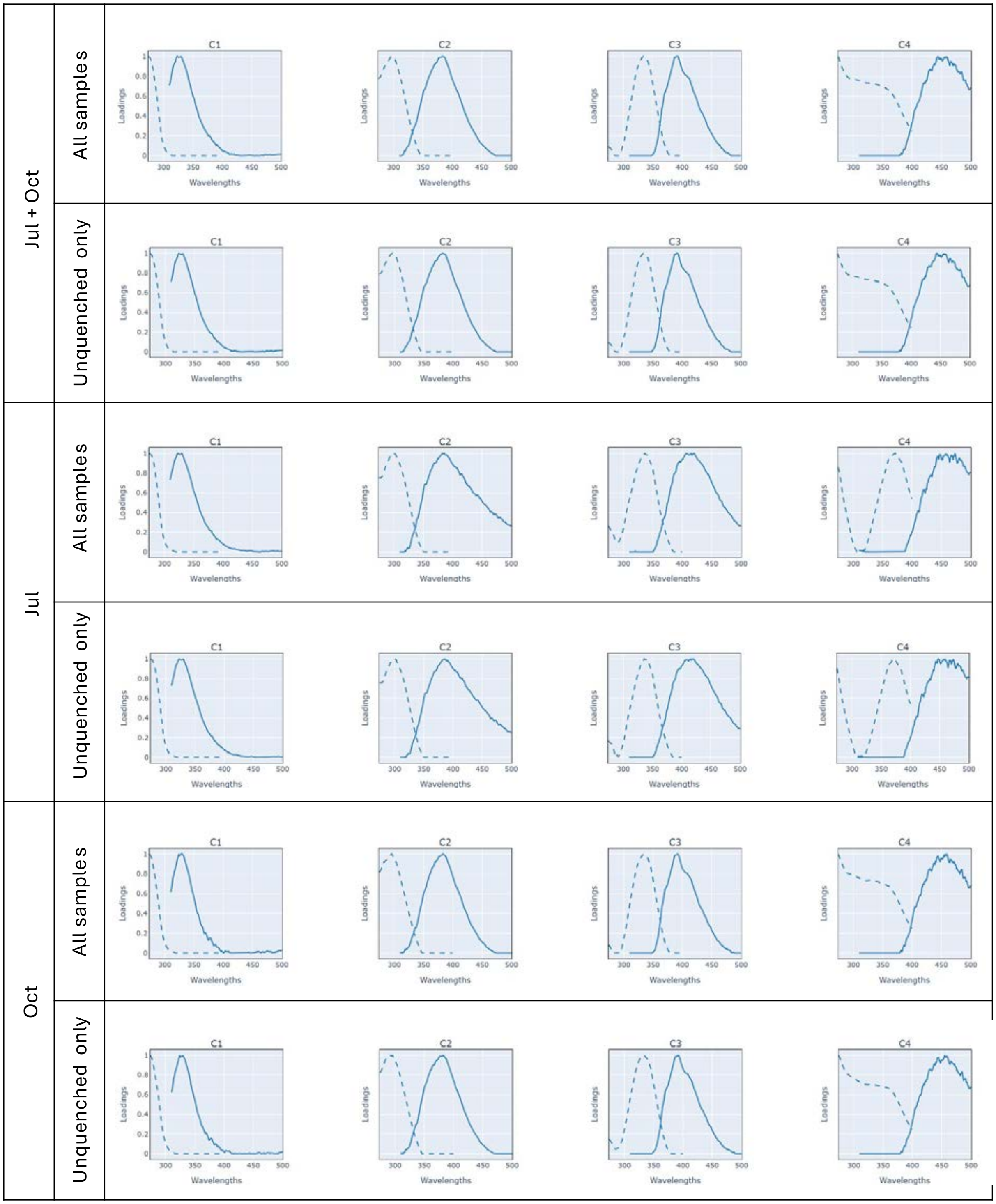

Figure S3. Excitation (dahsed line) and emission (solid line) loadings of PARAFAC models built on different dataset. “All samples” refer to unquenched + quenched samples.

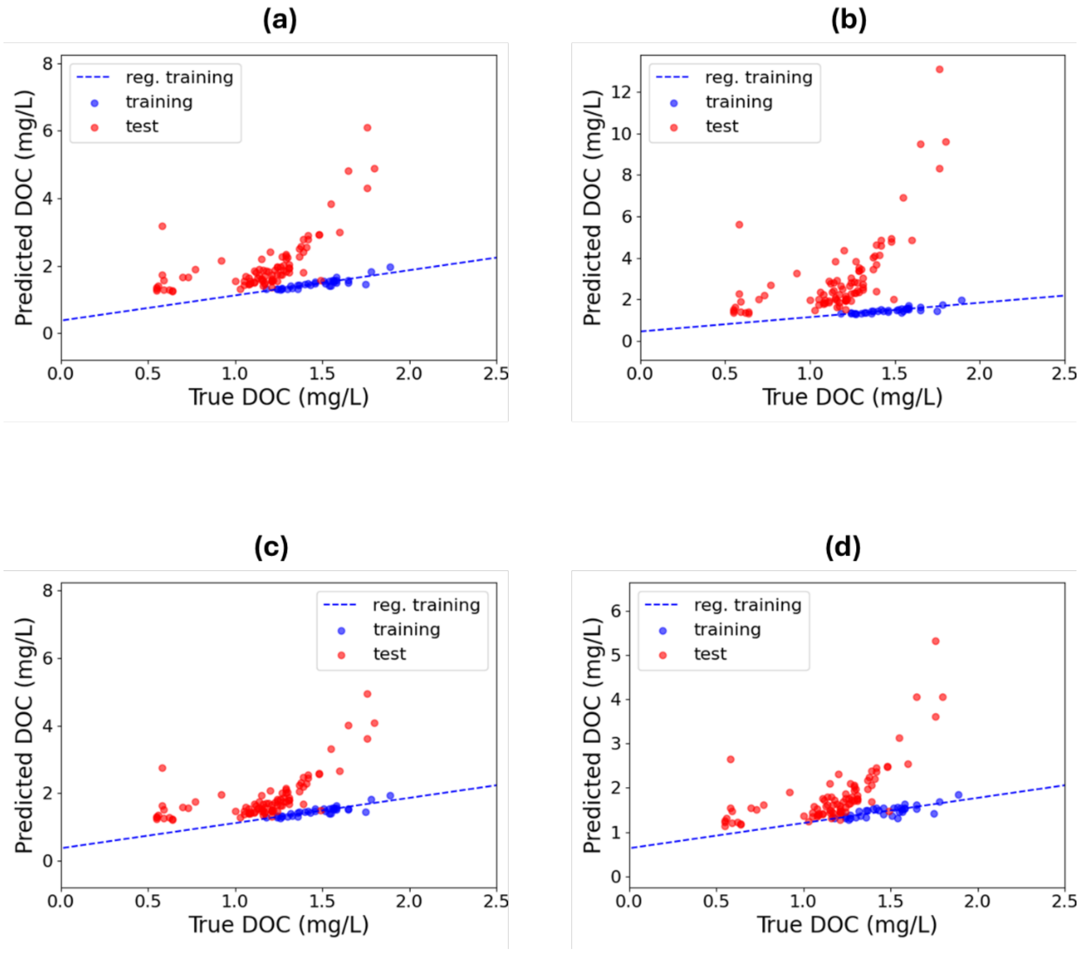

Figure S4. Training and testing of multivariate linear regression models using Fmax of multiple components. Both ordinary (a, c) linear regression and (b, d) non-negative regression with non-negative regression coefficients were tested. (a): using C2, C3 and C4 without non-negativity constraint; (b) using C1, C2 and C3 with non-negativity constraint; (c): using C1, C2, C3 and C4 without non-negativity constraint; (d) using C1, C2, C3 and C4 with non-negativity constraint.

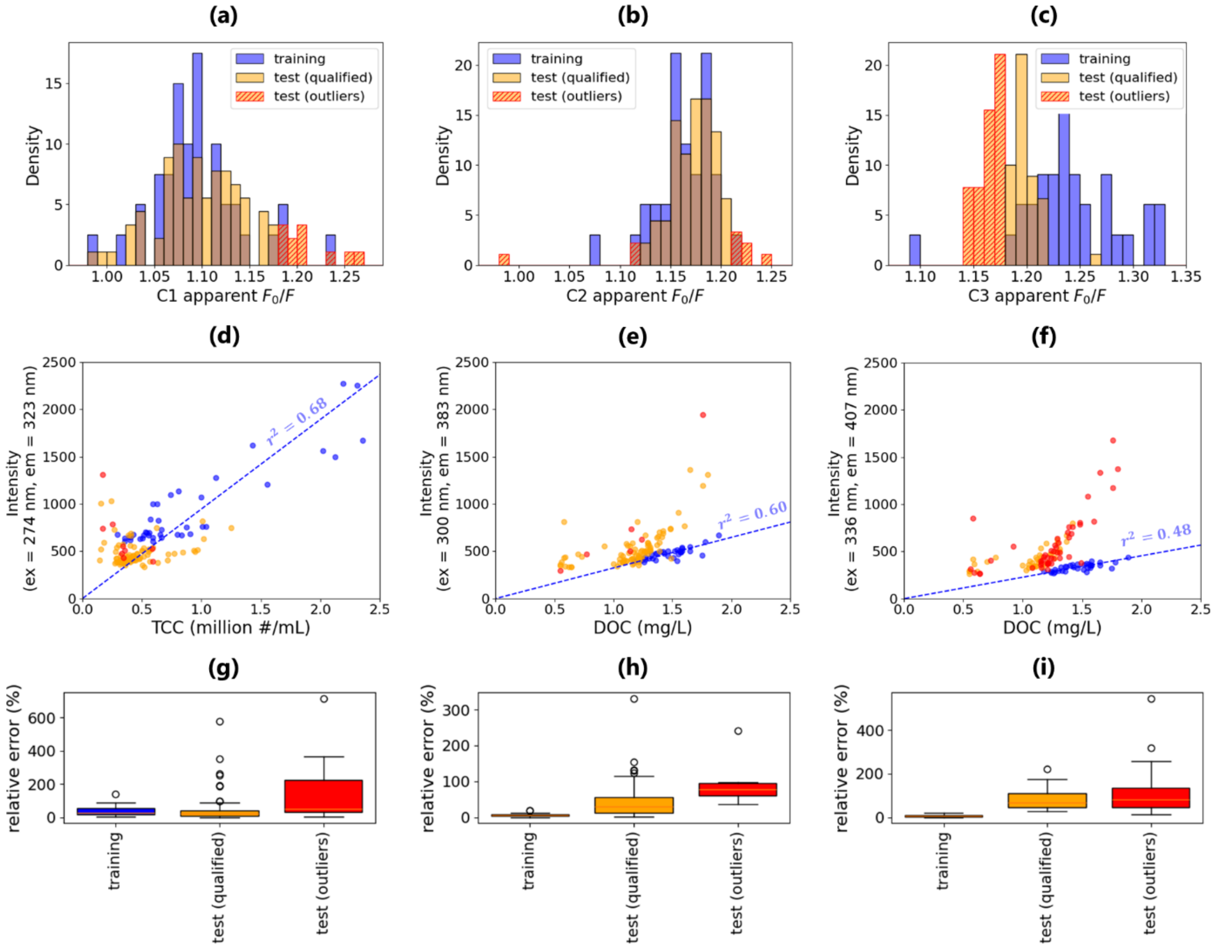

Figure S5. Performance of apparent F_0/F calculated from fluorescence intensities through peak-picking. The locations of the peaks were determined by the peaks of PARAFAC components C1, C2, and C3. The data in (a-i) are presented in the same form as Figure 4 in the main text.

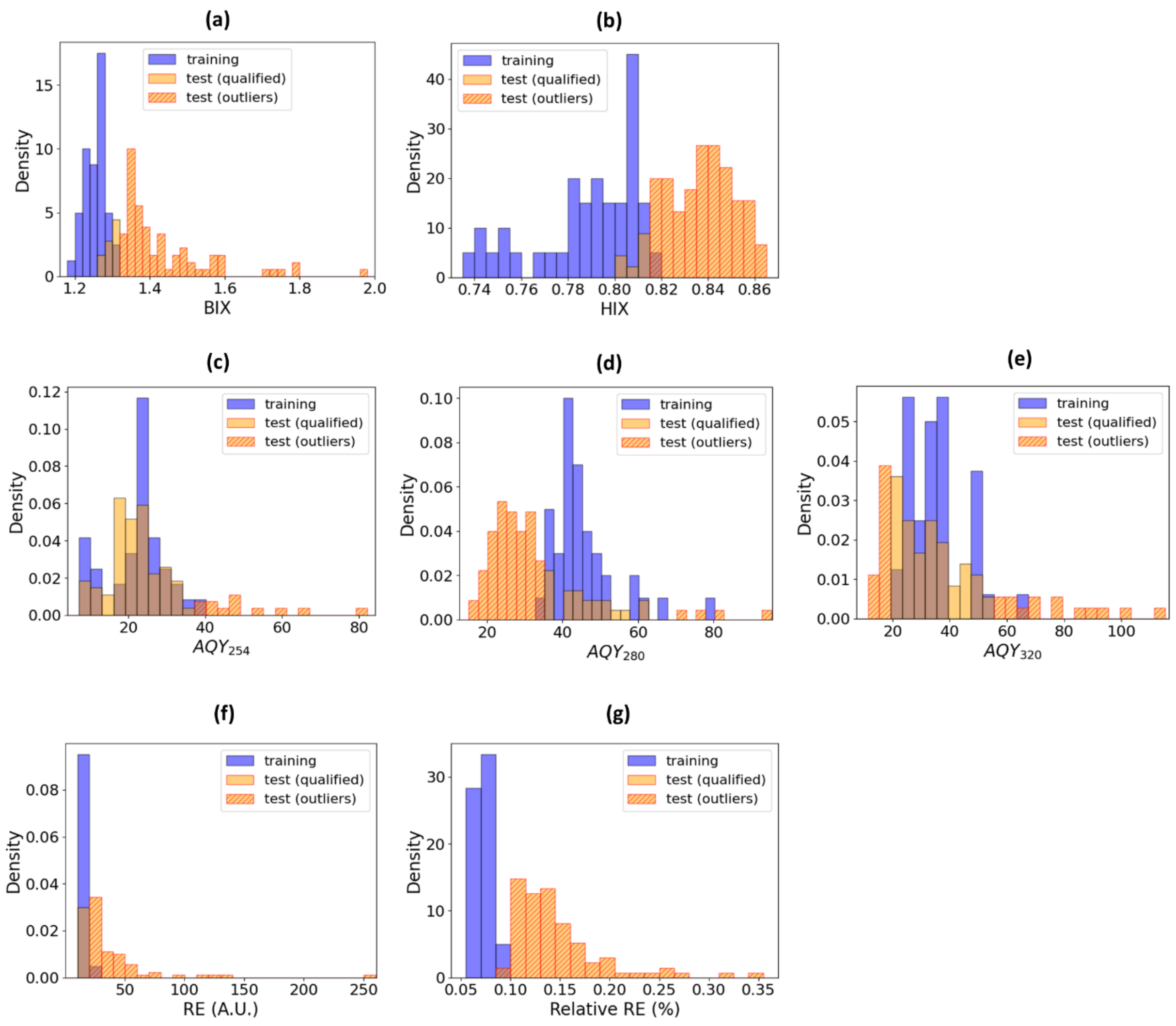

Figure S6. Density histograms of different fluorescence indices and error metrics. The PARAFAC model was trained on the July samples and tested on the October samples as described in chapter 3.4 in the main text. (a) BIX; (b) HIX; (c) AQY$_{254}$; (d) AQY$_{280}$; (e) AQY$_{320}$; (f) RE; (g) Relative RE.

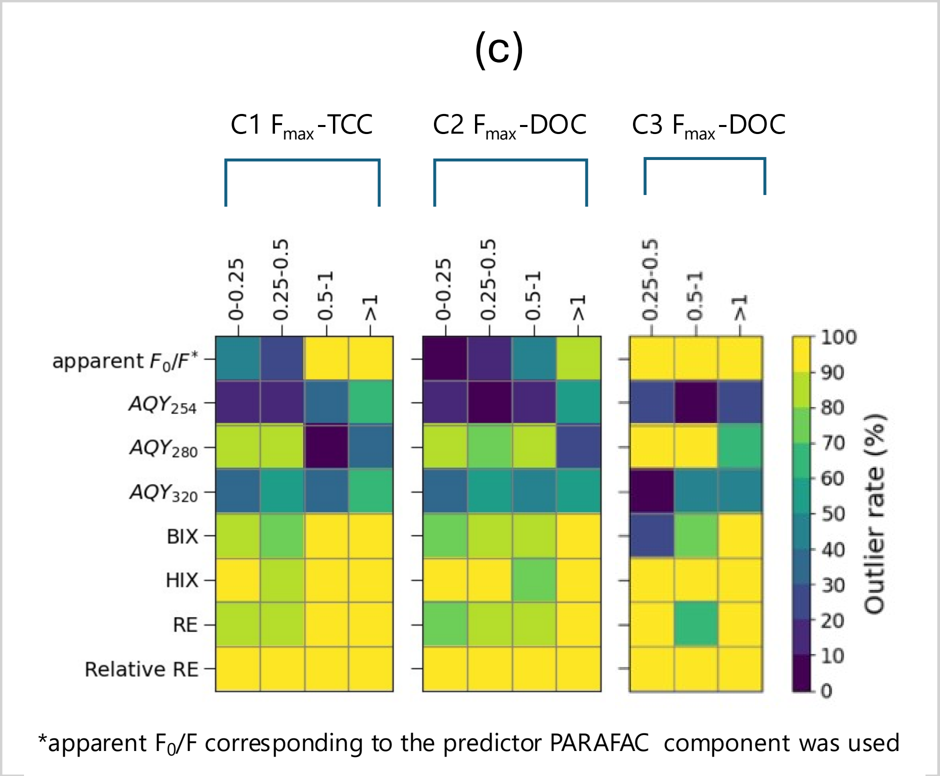

Figure S7. Outlier rates in model test given by different indicators (vertical axis) in absolute error categories (horizontal axis). (left) TCC prediction using C1 F_{max}, (middle) DOC prediction using C2 $F_{max,}$ and (right) DOC prediction using C3 F_{max}. The units of absolute errors are million #/mL, mg/L, and mg/L for C1, C2 and C3, respectively.

Supporting Tables

Table S1. Influent and effluent quality (25th, 50th and 75th quantiles) of the greywater reclamation system. Measurements were conducted from January 2024 to October 2024. Number of measurements for effluent TOC is 127, while other entries are 9.

**Location**	**TOC (mg/L)**	**NH_4-N (mg/L)**	**NO_3-N (mg/L)**
**Influent**	15.7, 19.8, 25.4	0.3, 0.6, 9.0	1.0, 2.0, 2.9
**Effluent**	1.16, 1.31, 1.56	<0.20	14.9, 15.4, 16.9

Table S2. Overview of PARAFAC models trained on different timescales using both unquenched and quenched samples. The r_{TCC} and r_{DOC} in the table come from the component Fmax with the highest Pearson correlation.

Dataset	Optimal number of components	Average split-half validation similarity	Best r^2 for TCC estimation[a]	Best r^2 for DOC estimation[a]
All (July + October)	4	0.98	0.43 (C1)	0.30 (C4)
July	4	0.93	0.70 (C1)	0.53 (C2)
October	4	0.99	-1.64 (C1)	0.47 (C3)

[a]Only accounts for relationships with positive slopes.

Table S3. Description of different fluorescence indices and EEM error metrics. All these indicators are calculated using original unquenched EEMs.

Indicator	Calculation	References
**Biological index (BIX)[a]**	$$\mathrm{BIX} = \frac{\mathrm{F}(310\ \mathrm{nm}, 380\ \mathrm{nm})}{\mathrm{F}(310\ \mathrm{nm}, 430\ \mathrm{nm})}$$ Where $\mathrm{F}(\lambda_{ex}, \lambda_{em})$ is the fluorescence intensity at excitation wavelength of λ_{ex} and emission wavelength of λ_{em}. This notation also applies to other equations below.	Zsolnay et al. (1999)[2]
**Humification index (HIX)[a]**	$$\mathrm{HIX} = \frac{\sum_{435\ \mathrm{nm}}^{480\ \mathrm{nm}} \mathrm{F}(254\ \mathrm{nm}, \lambda_{em})}{\sum_{300\ \mathrm{nm}}^{345\ \mathrm{nm}} \mathrm{F}(254\ \mathrm{nm}, \lambda_{em}) + \sum_{435\ \mathrm{nm}}^{480\ \mathrm{nm}} \mathrm{F}(254\ \mathrm{nm}, \lambda_{em})}$$	Huguet et al. (2009)[3]
**Apparent quantum yield (AQY)[a]**	$$\mathrm{AQY}_{\lambda_{ex}} = \frac{\sum_{280\ \mathrm{nm}}^{600\ \mathrm{nm}} \mathrm{F}(254\ \mathrm{nm}, \lambda_{em})}{\mathrm{A}(\lambda_{ex})}$$ Note that the calculation of AQY in this work did not apply normalization using a standard sample.	Wünsch et al. (2015)[4]
**Reconstruction error (RE)**	$$\mathrm{RE} = \sqrt{\frac{\sum_{i}^{N_{pixels}} (\mathrm{F}_{i,measured} - \mathrm{F}_{i,reconstructed})^2}{N_{pixels}}}$$ Where N_{pixels} refers to the number of total pixels in EEM.	Hu et al. (2025)[1]
**Relative reconstruction error (Relative RE)**	$$\mathrm{Relative\ RE} = \frac{\mathrm{RE}}{\frac{\sum_{i}^{N_{pixels}} \mathrm{F}_{i,measured}}{N_{pixels}}}$$	N/A

[a]Before the calculations of BIX, HIX and AQY, the EEMs were pre-processed using a 2-D gaussian filter with a bandwidth of 6 nm. The main purpose of this is to remove signal noise that could influence the fluorescence intensity at specific pixels. In addition, such pre-processing simulates signal from fluorescence sensors targeting at specific excitation and emission wavelengths, as the light sources of these sensors are often subject to specific wavelength bandwidths.

References

(1) Hu, Y.; Morgenroth, E.; Jacquin, C. Online Monitoring of Greywater Reuse System Using Excitation-Emission Matrix (EEM) and K-PARAFACs. *Water Res* **2025**, *268*, 122604. https://doi.org/10.1016/j.watres.2024.122604.

(2) Zsolnay, A.; Baigar, E.; Jimenez, M.; Steinweg, B.; Saccomandi, F. Differentiating with Fluorescence Spectroscopy the Sources of Dissolved Organic Matter in Soils Subjected to Drying. *Chemosphere* **1999**, *38* (1), 45–50. https://doi.org/10.1016/S0045-6535(98)00166-0.

(3) Huguet, A.; Vacher, L.; Relexans, S.; Saubusse, S.; Froidefond, J. M.; Parlanti, E. Properties of Fluorescent Dissolved Organic Matter in the Gironde Estuary. *Org Geochem* **2009**, *40* (6), 706–719. https://doi.org/10.1016/j.orggeochem.2009.03.002.

(4) Wünsch, U. J.; Murphy, K. R.; Stedmon, C. A. Fluorescence Quantum Yields of Natural Organic Matter and Organic Compounds: Implications for the Fluorescence-Based Interpretation of Organic Matter Composition. *Front Mar Sci* **2015**, *2* (NOV), 1–15. https://doi.org/10.3389/fmars.2015.00098.
